# Spectrum-Effect Relationships of Flavonoids in *Glycyrrhiza uralensis* Fisch.

**DOI:** 10.1155/2020/8838290

**Published:** 2020-12-03

**Authors:** Tingting Li, Shiyao Hua, Jiahua Ma, Lin Dong, Fang Xu, Xueyan Fu

**Affiliations:** ^1^Ningxia Medical University, Yinchuan 750004, China; ^2^Key Laboratory of Hui Ethnic Medicine Modernization, Ministry of Education (Ningxia Medical University), Yinchuan 750004, China

## Abstract

*Glycyrrhiza uralensis* Fisch. is used in large quantities in traditional Chinese medicine. It contains flavonoids, saponins, and polysaccharides, with flavonoids being the main active ingredients. In this study, flavonoids were isolated from the roots of *Glycyrrhiza uralensis* Fisch. grown in 21 areas in China by water extraction, alcohol precipitation, polyamide resin separation, and other methods. Fingerprints were established by high performance liquid chromatography (HPLC). There were 15 common peaks in the fingerprints by similarity evaluations of the chromatographic fingerprints. The spectrum-effect relationships between the HPLC fingerprints and pharmacological activities of flavonoids in *G. uralensis* Fisch., including the heat clearing, detoxifying effects, cough relief, and phlegm elimination effects, were assessed by gray relational analysis and partial least squares regression. After HPLC-quadrupole time-of-flight mass spectrometry and standard comparison, these five identified compounds (liquiritin apioside, neoisoliquiritin, licochalcone A, licochalcone B, and licochalcone C) could be used to evaluate licorice quality with regard to its efficacy. This research provides a scientific basis for improving licorice quality and also establishes a model for modernization of traditional Chinese medicines.

## 1. Introduction

Glycyrrhizae Radix et Rhizoma, the dried root and rhizome of *Glycyrrhiza uralensis* Fisch., *Glycyrrhiza inflata* Bat, or *Glycyrrhiza glabra* L. is commonly used in traditional Chinese medicine (TCM) [1] and described in the 2015 edition of the Chinese Pharmacopoeia. *Glycyrrhiza uralensis* Fisch. is one of the species included in the Pharmacopoeia [2]. It has a wide distribution and is planted over large areas in Northeast and Northwest China [3]. Licorice was first recorded in The Shennong Materia Medica and was described as having effects of invigorating the spleen and qi, clearing heat and detoxification, relieving coughing and eliminating phlegm, relieving spasms and pain, and mediating the effects of various medicines [3]. In modern pharmacological studies, many active components of licorice have been observed to have a range of pharmacological activities, including hypocholesterolemic, hypoglycemic, anxiolytic, antimicrobial, antiviral, free radical scavenging, antiulcer, cytotoxic, antitumor, antiallergic, antidiabetic, anticarcinogenic, antioxidant, anti-inflammatory, and hepatoprotective activities. Licorice is widely used in food, medicines, the chemical industry, and other fields [4, 5]. At present, traditional formulations of licorice from other countries, such as Japan, Iran, and South Korea, are also widely used [6–8]. It is produced in China and is sold in many countries worldwide. Comprehensive development and utilization of licorice resources are expanding; then the market prospects are very broad. The content of total flavonoids and specific flavonoids in licorice from different sources varies greatly [6–8].

According to the 2015 edition of the Chinese pharmacopoeia, there are limitations to controlling glycyrrhizin and liquiritin. However, we do not know if their content can reflect their clinical utility and can be used to control the quality of licorice. Therefore, it is of great significance to establish a method to evaluate the complexity and integrity of licorice and improve quality standards. Current evaluation models lead to neglect of the interaction effects of multicomponent and multitarget TCMs and do not allow for optimum quality control of licorice. Hence, it is important to establish a method to evaluate complex samples such as licorice.

The spectrum-effect relationship is an effective method to evaluate the quality of TCMs [9–11]. Spectrum-effect relationship studies focus on correlations between fingerprint characteristics and pharmacodynamic data. The qualities of TCMs are evaluated using chemical components and biological activity, and the relative contributions of different components to the efficacy are determined. The results can be used to identify components that are most closely related to the pharmaceutical effect and accurately reflect the quality of a TCM.

In this study, the chromatographic fingerprints of 21 different producing areas of licorice were obtained using HPLC following existing quality control methods for crude TCMs. Common peaks were determined using similarity evaluation system software to create chromatographic fingerprints. The compounds for the selected common peaks were identified by HPLC-quadrupole time-of-flight (Q-TOF) mass spectrometry (MS) and comparison with standard samples. Data from experiments investigating the heat clearing and detoxification effects and cough relief and phlegm elimination effects were used to establish spectrum-effect relationships that were assessed using gray relational analysis (GRA) and partial least squares (PLS) regression. Flavonoid components of licorice could be used as indicators for quality control of licorice which were connected with the traditional effects identified. This research provides a method and scientific data for comprehensively improving licorice quality control.

## 2. Experimental

### 2.1. Reagents and Experimental Animals

Twenty-one production areas of dried licorice (Table 1) were collected from different growing areas in China, including Ningxia, Gansu, Inner Mongolia, Xinjiang, and Heilongjiang, in 2017, and identified by professor Zhang Xinhui at Ningxia Medical University (Yinchuan, China). Licorice standards (Table 2) were purchased from Yuanye Biotechnology Co. Ltd. (Shanghai, China). We used the following solvents in the experiments: acetonitrile (purity = 100%, HPLC-grade; Thermo Fisher Scientific, Waltham, MA, USA); glacial acetic acid (purity = 100%, HPLC grade; Kaixin Chemical Industry Co. Ltd, Tianjin, China); HPLC grade methanol, ethanol, and analytical grade ethanol (purity = 95%, HPLC grade); and xylene and other reagents (Damao Chemical Reagent Production, Tianjin, China). Specific pathogen free-grade ICR male mice weighing 18–22 g were provided by the experimental animal center at Ningxia Medical University (animal certificate No. SCXK (Ning) 2015-0001). The mice were raised at room temperature (22°C ± 2°C) with a relative humidity of 50%–60% and natural light. For the experiments involving mice, they were randomly divided into a model group, blank group, and licorice treatment group with 10 mice in each group. Disease was induced in the model group but not the blank group, and both groups were given sodium carboxymethyl cellulose (CMC-Na) aqueous solution. Mice in the treatment group were continuously administered 150 mg/kg licorice total flavonoids for 1 week, and those in the blank and model groups were fed CMC-Na once per day. The blank group was not subjected to any experimental treatment.

### 2.2. Instrumentation and Chromatography Condition

#### 2.2.1. Chromatography Condition

The chromatographic separation was performed on a ZORBAX SB-C_18_ Column (250 mm × 4.6 mm, 5 *μ*m; Agilent Technologies) maintained at 30°C and using a diode array detector (DAD) with the detection wavelength set to 310 nm. The mobile phase was a mixture of 0.2% glacial acetic acid (A) and acetonitrile (B) with a flow rate of 1.0 mL/min. The sample injection volume was 20 *μL* and the sample was separated using the following optimized gradient elution: 0–15 min, 15%B; 15–25 min, 15–20% B; 25–70 min, 20–50% B; 70–90 min, 50–70% B; and 90–95 min, 70–15% B. The results were analyzed using Agilent Chem Station.

MS measurements were performed with a 6545 Q-TOF instrument equipped with an electrospray ionization source (Dual AJS ESI+, Agilent Technologies). The drying gas temperature and source temperature were maintained at 350°C and 120°C, respectively. The MS capillary voltage, cone voltage, and frag mentor voltage were fixed at 3500 V, 30 V, and 130 V, respectively. The drying gas flow rate was 10 L/min and the nebulizer pressure was 40 psi. The mass scan range was 50–1000 m/z.

#### 2.2.2. Methodological Validation

One sample solution was analyzed six times to determine the precision. The RSD values of the peak area of each major chromatograph were all less than 3.5%, and the RSD values of the relative retention time were all less than 1.8%, indicating good precision of the instrument. A stability study was performed by analyzing a sample at different intervals over 1 day (0, 2, 4, 8, 12, and 24 h). The RSD values of the peak-peak area of each major chromatograph were all less than 3.0%, and the RSD values of the relative retention time were all less than 2.0%, indicating that the sample solution was stable within 24 h. Six sample solutions from the same batch were analyzed to determine the repeatability. The analysis of each sample was repeated three times. The RSD values of each main chromatographic peak area were all less than 5.0%, and the RSD values of retention time were all less than 2.4%, indicating that the method has good repeatability.

Then standard solution of the individual component was diluted gradually, to determine its limit of detection (LOD) values were determined by using signal-to-noise ratios of 10 : 1, as follows: liquiritin, 0.035 *μ*g/mL; isoliquiritin, 0.047 *μ*g/mL; liquiritigenin, 0.032 *μ*g/mL; isoliquiritigenin, 0.044 *μ*g/mL; liquiritin apioside, 0.025 *μ*g/mL; isoliquiritin apioside, 0.053 *μ*g/mL; neoliquiritin, 0.033 *μ*g/mL; neoisoliquiritin, 0.041 *μ*g/mL; glabridin, 0.058 *μ*g/mL; licochalcone A, 0.108 *μ*g/mL; licochalcone B 0.076 *μ*g/mL; licochalcone C, 0.012 *μ*g/mL; formononetin, 0.034 *μ*g/mL; glycyrrhizic acid, 0.056 *μ*g/mL; and *β*-glycyrrhetinic acid, 0.065 *μ*g/mL.

### 2.3. The Total Flavonoids Content

#### 2.3.1. Preparation of Liquiritin Standard Solution

Liquiritin was precisely weighed and dissolved with 70% ethanol in a 10 mL volumetric flask to prepare a solution with a concentration of 1.05 mg/mL.

#### 2.3.2. Preparation of Sample Solutions

Samples of the licorice flavonoids concentrates were weighed to 30 mg in 10 mL volumetric flasks and 70% ethanol was added. After ultrasonication for 30 mins, each sample was weighed and then stored at 4°C until required for analysis.

#### 2.3.3. Standard Curve of Liquiritin

The linearity was studied by analyzing liquiritin standard solutions with five different concentrations and determining the absorbance by UV spectrophotometry at 213 nm (after full wavelength scanning, there is the maximum absorption wavelength). Taking the absorbance value (*A*) as the *x*-axis, the concentrations of liquiritin were plotted on the *y*-axis to construct standard curves. The regression equation was *y* = 1.1299*x* + 3.2989. The linear range was 0.021–0.105 mg/mL and the correlation coefficient was higher than 0.999.

#### 2.3.4. Determination of Total Flavonoids of 21 Production Areas

Preparation of sample solutions and method validation for quantitative analysis of total flavonoids content were stated in the supplemental material. The absorbance values for solutions of licorice total flavonoids from the 21 growing areas were determined using the method described in Section 2.3.3.

### 2.4. HPLC Fingerprints

#### 2.4.1. Preparation of Sample Solutions

Preparation method was described in Section 2.3.2.

#### 2.4.2. Preparation of a Mixed Standard Solution

Individual stock solutions at the following concentrations were prepared by dissolving standards in 70% ethanol: liquiritin, 4.07 *μ*g/mL; isoliquiritin, 4.07 *μ*g/mL; liquiritigenin, 3.38 *μ*g/mL; isoliquiritigenin, 4.15 *μ*g/mL; liquiritin apioside, 4.00 *μ*g/mL; isoliquiritin apioside, 3.84 *μ*g/mL; neoliquiritin, 3.69 *μ*g/mL; neoisoliquiritin, 3.08 *μ*g/mL; glabridin, 3.30 *μ*g/mL; licochalcone A, 3.54 *μ*g/mL; licochalcone B 3.08 *μ*g/mL; licochalcone C, 1.92 *μ*g/mL; formononetin, 3.69 *μ*g/mL; glycyrrhizic acid, 3.69 *μ*g/mL; and *β*-glycyrrhetinic acid, 3.69 *μ*g/mL. Take 100 microliters of each standard solution and mix them together to make a mixed standard solution. After ultrasonication, the prepared solutions were stored at 4°C until required for use.

#### 2.4.3. Similarity Evaluation of the Fingerprints

HPLC results for the samples from the 21 growing areas, including the retention times and peak areas, were exported in AIA (∗.cdf) format in Chinese Medicine Fingerprint Similarity Evaluation (2004A Version). The median method is used to obtain the result. A representative reference fingerprint was automatically constructed using the median method by comparing the results for the 21 producing areas of licorice flavonoid extracts. Similarity values between the chromatogram for each batch and the reference fingerprint were calculated using software. Then, the results were used to identify common peaks for the licorice flavonoids.

#### 2.4.4. Identification of Common Peaks

Under the conditions described in Section 2.4.2, the mixed standard and sample solutions were analyzed by HPLC-Q-TOF/MS. The spectrum of the mixed standard sample was compared with those of the sample solutions, and common peaks were identified according to the retention times and fragment ions. The flavonoid components for these peaks were inferred.

### 2.5. Cough Relief and Phlegm Elimination Effects

#### 2.5.1. Phenol Red Excretion in Mice Trachea

One hour after the last dose, the mice were intraperitoneally injected with a 5% phenol red normal saline solution. After 30 min, the mice were killed by dislocation. The trachea is separated and flushed with 0.5 mL of a 5% sodium bicarbonate (NaHCO_3_) solution [9–11]. We combine the flushing solution into a small test tube and centrifuge at 2000 rpm for 10 min.

#### 2.5.2. Ammonia-Induced Cough Test

Thirty minutes after the last treatment, the mice in the model and treatment groups were exposed to ammonia gas in a 500 mL beaker. The number of times the mice coughed in 2 min was recorded, with abdominal muscle contractions and yawning taken as coughing.

#### 2.5.3. Sulfur Dioxide-Induced Cough Test

After the last dose, the mice were exposed to sulfur dioxide in a bell jar. For each group, the cough incubation period and the number of times the mice coughed within 3 min were observed and recorded [12].

### 2.6. Heat Clearing and Detoxification Effects

#### 2.6.1. Mouse Acute Paw Swelling Tests

After the last dose, 0.1 mL of a 1% carrageenan solution was subcutaneously injected into the plantar area of each mouse [13]. The degree of swelling was calculated at 0, 1, 2, 3, 4, and 5 h using the following equation: degree of swelling = foot volume at the measurement time − foot volume at 0 h.

#### 2.6.2. Mouse Acute Ear Swelling Test

After the last dose, the left ears of all the mice were uniformly coated with xylene, except for those in the blank group, which were coated with distilled water. The mice were euthanized 1 h after the ears were coated. The left and right ears of the mice were cut off, and pieces of the ear were taken from the same position with an 8 mm perforator and weighed, and the degree of swelling was calculated using the following equation: swelling = left ear mass − right ear mass.

### 2.7. Data Statistics

The experimental data are expressed as the means ± SDs. The LSD and Dunnett's T3 test (3) were used for intergroup comparisons.

### 2.8. Statistical Analysis of the Spectrum-Effect Relationships

Correlation analysis was conducted between the common peak areas and pharmacodynamic data of licorice flavonoids from different regions using the HPLC data by GRA and PLS method. SPSS 24.0 (International Business Machines Corporation, New York, USA) and DPS 7.05 (Zhejiang University, Hangzhou, China) were used to process the data and find common peaks that were significantly related to the pharmacological effects.

## 3. Results and Discussion

### 3.1. Determination of the Total Flavonoids Content in Licorice

**Figure 1:**
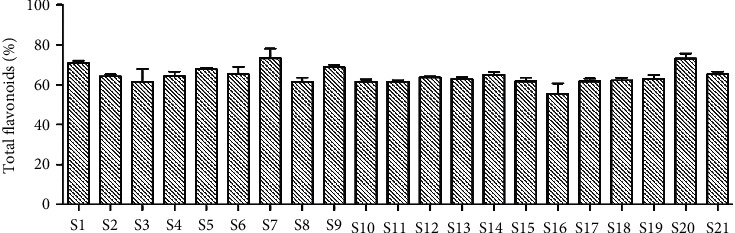
Determination of flavonoids in *Glycyrrhiza uralensis* Fisch. from 21 producing areas by UV. The result represents mean ± S.D.

The total flavonoids contents in the 21 samples from different growing areas were calculated from the UV spectrophotometry results (Figure 1). The content of total flavonoids of licorice root was higher than 60% except S16. The content of licorice flavonoids was the highest in S7. Licorice has a complex chemical composition [4, 14]. Flavonoids are one of the main active substances in licorice. Modern pharmacological studies have shown that it has multiple activities [15]. Studies have shown that the contents and types of licorice flavonoids from different sources vary greatly [16]. Licorice flavonoids are closely related to the traditional effects of licorice in TCM. In this study, the enrichment method can obtain relatively reliable licorice flavonoids and provide sufficient guarantee for further research.

### 3.2. Chromatographic Fingerprints and Similarity Analysis

**Figure 2:**
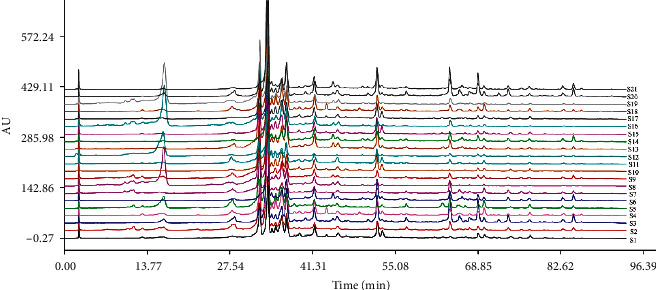
HPLC fingerprint of total flavonoids of *Glycyrrhiza uralensis* Fisch. from 21 producing areas.

**Figure 3:**
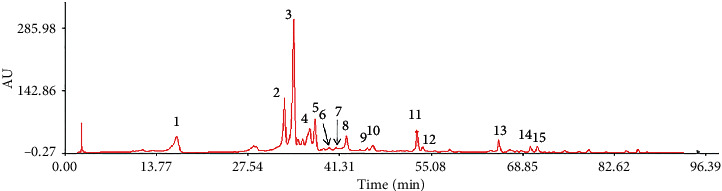
HPLC fingerprint of total flavonoids of *Glycyrrhiza uralensis* Fisch. from 21 producing areas.

The 21 licorice samples were injected into the HPLC and chromatograms were recorded. The results are shown in Figure 2. The spectrum of sample S1 was selected as the reference spectrum, and the software automatically matched the chromatographic peaks through multipoint correction. A control fingerprint (Figure 3) was generated by the selected median method, and 15 common peaks were analyzed. The similarity value of the generated control fingerprint (R) was set as one, and the similarities of the characteristic chromatograms of the 21 licorice samples were calculated. The origins of the licorice samples and fingerprint similarities are shown in Table 3. Sixteen samples had similarities greater than 0.9. We suspected that this difference between those samples could be caused by different producing areas of the licorice.

### 3.3. Pharmacodynamic Experiments

#### 3.3.1. Test Results for Cough Relief and Phlegm Elimination

**Figure 4:**
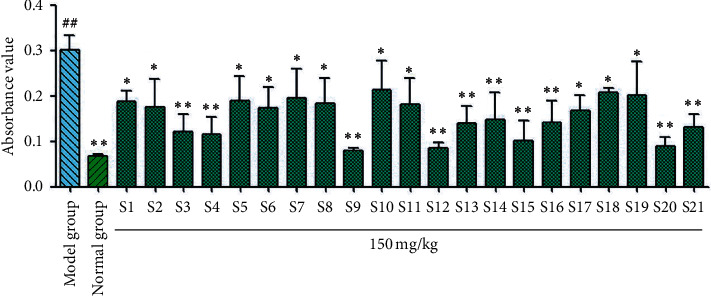
Results of tracheal phenol red of flavonoids in *Glycyrrhiza uralensis* Fisch. from 21 producing areas. The result represents mean ± S.D. *Note.* Compared with the normal group, ^##^*P* < 0.01 is a very significant difference. Compared with the model group, ^*∗*^*P* < 0.05 was considered as significant difference; ^*∗∗*^*P* < 0.01, and there was a significant difference (*n* = 10).

**Figure 5:**
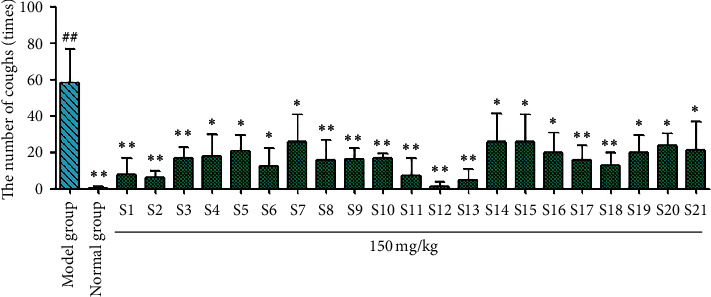
Results of ammonia-induced cough in *Glycyrrhiza uralensis* Fisch. flavonoids from 21 producing areas. The result represents mean ± S.D. *Note.* Compared with the normal group, ^##^*P* < 0.01 is a very significant difference. Compared with the model group, ^*∗*^*P* < 0.05 was considered to be significant; ^*∗∗*^*P* < 0.01, and there was a significant difference (*n* = 10).

**Figure 6:**
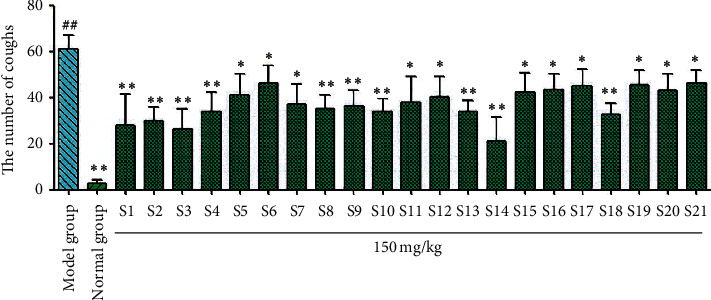
Results of SO_2_-induced cough in *Glycyrrhiza uralensis* Fisch. flavonoids from 21 producing areas. The result represents mean ± S.D. *Note.* Compared with the normal group, ^##^*P* < 0.01 is a very significant difference. Compared with the model group, ^*∗*^*P* < 0.05 was considered to be significant; ^*∗∗*^*P* < 0.01, and there was a significant difference (*n* = 10).

According to the tracheal phenol red experiment results (Figure 4), phenol red excretion in the model group was significantly reduced (^##^*P* < 0.01) compared with the blank group, indicating successful modeling. Compared with the model group, the licorice flavonoids from samples S1–S21 significantly reduced the excretion of tracheal phenol red (^*∗*^*P* < 0.05 and ^*∗∗*^*P* < 0.01). The results of the ammonia cough test (Figure 5) showed that the total licorice flavonoids decreased the frequency of coughing in mice significantly compared with the model group, but to different degrees (^*∗*^*P* < 0.05 and ^*∗∗*^*P* < 0.01). The licorice flavonoids had a therapeutic effect on coughing caused by ammonia. Samples S1, S2, S3, S8, S9, S10, S11, S12, S13, S17, and S18 significantly reduced coughing. For the sulfur dioxide cough test (Figure 6), compared with the model group, the licorice flavonoids reduced the frequency of coughing to different degrees (^*∗*^*P* < 0.05, ^*∗∗*^*P* < 0.01). Samples S1, S2, S3, S4, S8, S9, S10, S13, S14, and S18 showed the greatest reductions in coughing, whereas the antitussive effects of samples S5, S6, S7, S11, S12, S15, S16, S17, S19, S20, and S21 were slightly weaker.

#### 3.3.2. Test Results for Heat Clearing and Detoxification

**Figure 7:**
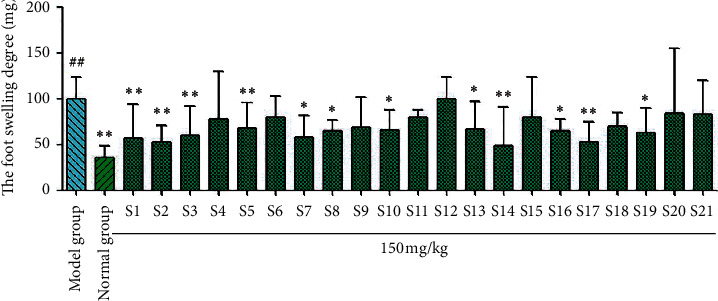
Results of foot swelling of *Glycyrrhiza uralensis* Fisch. flavonoids from 21 producing areas. The result represents mean ± S.D. *Note.* Compared with the normal group, ^##^*P* < 0.01 is a very significant difference. Compared with the model group, ^*∗*^*P* < 0.05 was considered to be significant; ^*∗∗*^*P* < 0.01, and there was a significant difference (*n* = 10).

**Figure 8:**
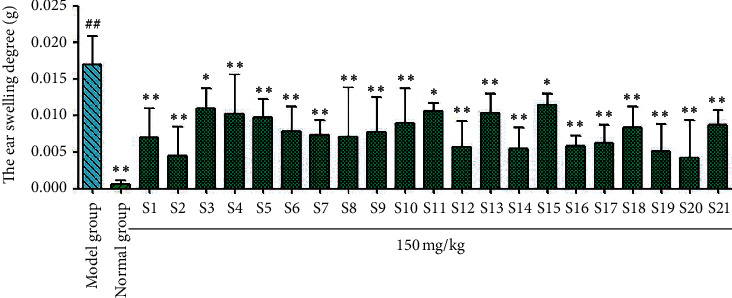
Results of ear swelling of *Glycyrrhiza uralensis* Fisch. flavonoids from 21 producing areas. The result represents mean ± S.D. *Note.* Compared with the normal group, ^##^*P* < 0.01 is a very significant difference. Compared with the model group, ^*∗*^*P* < 0.05 was considered as significant; ^*∗∗*^*P* < 0.01, and there was a significant difference (*n* = 10).

The results of carrageenan-induced mouse paw swelling tests (Figure 7) showed that paw swelling in mice treated with licorice samples S1, S2, S3, S5, S7, S8, S10, S13, S14, S16, S17, and S19 was significantly (^*∗*^*P* < 0.05 and ^*∗∗*^*P* < 0.01) lower than that in the model group. The licorice flavonoids in the other samples did not significantly affect paw swelling in mice (*P* > 0.05). For the xylene-induced ear swelling in mice (Figure 8), the licorice flavonoids from all 21 samples significantly reduced ear swelling (^*∗*^*P* < 0.05 and ^*∗∗*^*P* < 0.01) compared with the model group.

### 3.4. Spectrum-Effect Relationship Results

#### 3.4.1. Cough Relief and Phlegm Elimination Effects

With GRA Method, 14 peaks were identified with correlation degrees greater than 0.5 in the results of the tracheal phenol red experiment (Table 4). The order of the correlation degrees of these 14 peaks was 8 > 9 > 14 > 12 > 15 > 6 > 4 > 3 > 5 > 13 > 7 > 11 > 10 > 2. These peaks were closely related to the efficacy of licorice with regard to eliminating phlegm. From the ammonia exposure results (Table 5), 14 peaks were identified to have correlation degrees of greater than 0.5 with the frequency of coughing in mice. The contributions of these peaks were in the order 8 > 14 > 4 > 3 > 5 > 12 > 9 > 7 > 6 > 13 > 11 > 7 > 1 > 10 > 15. These peaks were closely related to the efficacy of licorice with regard to coughing induced by ammonia. From the results of the sulfur dioxide cough tests in mice (Table 6), correlation degrees of 14 peaks were more than 0.5. These peaks were in the order 15 > 9 > 14 > 12 > 5 > 4 > 8 > 3 > 11 > 7 > 6 > 10 > 13 > 1. These peaks were closely related to the efficacy of licorice with regard to coughing induced by sulfur dioxide.

With the PLS regression method, the regression coefficients reflected the contribution of each *x* to *y*. The larger the absolute value of the PLS regression coefficient, the greater the contribution of the peak to medical efficacy. Peaks were negatively correlated with the absorbance; that is, as the peak intensity increased, the absorbance value decreased. The remaining peaks were positively correlated with the absorbance. The pharmacodynamic experiment results of the tracheal phenol red test and the common peak areas were fitted by the regression equation *y* = 0.0348 *x*1 − 0.1386 *x*2 + 0.0808 *x*3 + 0.0890 *x*4 − 0.1985 *x*5 + 0.0940 *x*6 − 0.0079 *x*7 + 0.0749 *x*8 + 0.1659 *x*9 − 0.0857 *x*10 − 0.1424 *x*11 + 0.0952 *x*12 + 0.0280 *x*13 + 0.0136 *x*14 + 0.0661 *x*15. In this regression equation, the contribution rates of the 15 common peak were in the order 5 > 9 > 11 > 2 > 12 > 6 > 4 > 3 > 10 > 8 > 15 > 1 > 13 > 14 > 7. For the ammonia cough test results, the regression equation from PLS regression analysis was *y* = 0.0055 *x*1 + 0.0861 *x*2 + 0.0127 *x*3 − 0.0234 *x*4 + 0.0875 *x*5 + 0.0639 *x*6 − 0.0126 *x*7 + 0.0123 *x*8 − 0.0033 *x*9 + 0.0170 *x*10 + 0.0540 *x*11 − 0.0085 *x*12 + 0.0023 *x*13 + 0.1469 *x*14 + 0.1137 *x*15. The contribution rates of the 15 common peaks were in the order 14 > 15 > 5 > 2 > 6 > 11 > 4 > 10 > 3 > 7 > 8 > 12 > 1 > 9 > 13. The other peaks were positively correlated. For the mice sulfur dioxide coughing test results, the regression equation was *y* = 0.0054 *x*1 − 0.0244 *x*2 − 0.1618 *x*3 − 0.0506 *x*4 + 0.0741 *x*5 − 0.1443 *x*6 + 0.0460 *x*7 + 0.0101 *x*8 + 0.0269 *x*9 − 0.0297 *x*10 + 0.0608 *x*11 − 0.0081 *x*12 − 0.1336 *x*13 − 0.1060 *x*14 − 0.0805 *x*15. The contribution rates of the 15 common peaks were in the order 3 > 6 > 13 > 14 > 15 > 5 > 11 > 1 > 4 > 7 > 10 > 9 > 2 > 8 > 12.

Considering the results from the GRA and PLS methods, eight chromatographic peaks (3, 4, 5, 6, 9, 12, 14, and 15) were identified as being correlated with cough relief and phlegm elimination effects.

#### 3.4.2. Heat Clearing and Detoxification Effects

With GRA method, the results of the carrageenan-induced paw swelling experiments in mice showed that 14 peaks had correlation degrees of greater than 0.5 with swelling (Table 7). The correlation degrees of these peaks were in the order 8 > 12 > 4 > 14 > 5 > 15 > 3 > 6 > 7 > 9 > 11 > 13 > 10 > 1. These peaks were closely related to the efficacy of the drug with regard to the reduction of paw swelling. The results of the xylene-induced ear swelling experiments in mice showed that 14 peaks had correlation degrees of greater than 0.5 with ear swelling (Table 8). The correlation degrees of these peaks were in the order 8 > 15 > 5 > 3 > 14 > 4 > 6 > 9 > 12 > 11 > 7 > 13 > 2 > 10. These peaks were closely related to the efficacy of the drug with regards to the reduction of ear swelling.

With PLS regression method, the carrageenan-induced paw swelling results gave the regression equation *y* = 0.0017 *x*1 − 0.0178 *x*2 − 0.1593 *x*3 − 0.0411 *x*4 + 0.0770 *x*5 − 0.0789 *x*6 + 0.0948 *x*7 − 0.0670 *x*8 − 0.0617 *x*9 + 0.0511 *x*10 + 0.0838 *x*11 + 0.0516 *x*12–0.0423 *x*13 − 0.0731 *x*14 − 0.0558 *x*15. In this regression equation, the contribution rates of the 15 common peaks were in the order 3 > 7 > 11 > 6 > 5 > 14 > 8 > 9 > 15 > 12 > 10 > 13 > 4 > 2 > 1. PLS regression of the xylene-induced ear swelling results gave the regression equation *y* = −0.1294 *x*1 + 0.0153 *x*2 + 0.0687 *x*3 + 0.0248 *x*4 + 0.0140 *x*5 + 0.0827 *x*6 + 0.0346 *x*7 + 0.1777 *x*8 + 0.0692 *x*9 + 0.0648 *x*10 + 0.0646 *x*11 + 0.0687 *x*12 + 0.0886 *x*13 + 0.0761 *x*14 + 0.1347 *x*15. The contribution rates of the 15 common peaks were in the order 8 > 15 > 1 > 6 > 13 > 14 > 9 > 3 > 12 > 10 > 11 > 7 > 4 > 2 > 5. Considering the results from the two methods comprehensively, seven chromatographic peaks (3, 5, 6, 7, 8, 14, and 15) were identified as contributing to the heat clearing and detoxification effects.

Licorice has attracted the attention of many researchers in recent decades. Many chromatographic techniques have been applied to licorice quality control [1, 17]. Liu et al. used a single standard to quantify eight important active markers in licorice. The easily available glycyrrhizic acid was selected as a reference substance to calculate relative response factors [18]. These studies have improved the quality of licorice. Based on previous studies, we tried to use the Spectrum-effect relationship between HPLC fingerprints and effect to find the chemical components related to the efficacy and use them as the quality evaluation criteria. The modern pharmacology research models selected in this study are representative of traditional models for evaluating efficacy. The main data analysis and processing methods currently available for spectrum-effect research are GRA and PLS regression. The GRA method can describe the size, strength, and order of factors using the gray correlation order. This can be used to determine the degree of influence of different factors or the contributions of different factors to the main effects [19]. PLS regression is an effective method to study spectrum-effect relationships in TCMs [13, 20]. The correlation degree can be evaluated using the correlation coefficient.

In this study, GRA and PLS regression analysis were applied to study the spectrum-effect relationships of the 21 licorice samples. The fingerprint and pharmacological data were analyzed to determine what peaks were closely related to the licorice efficacy. The important components of licorice were identified according to their contributions to the studied pharmacological effects. Finally, five flavonoids (peaks 3, 5, 6, 14, and 15) were identified as being closely related to the cough relief and phlegm elimination effects and heat clearing and detoxification effects.

### 3.5. Identification of Common Peaks

According to literature reports and expected cleavage of licorice compounds from PubChem and Sci Finder, a licorice database was established. Common peaks of the mixed standard solution and test solutions were subjected to HPLC-Q-TOF/MS in positive ion mode under the above conditions (Section 2.4.2). The analysis and matching were conducted by Agilent Mass Hunter Qualitative Analysis software using reference data, mass spectrometry data, and chromatographic retention data from the literature [21]. After this comparison, the following components were identified: liquiritin, isoliquiritin apioside, isoliquiritin, neoisoliquiritigenin, licochalcone B, isoliquiritigenin, liquiritigenin, formononetin, licochalcone C, and licochalcone A. The five peaks (3, 5, 6, 14, and 15) in the flavonoids fingerprint were closely related to its traditional efficacy, including liquiritin apioside, neoisoliquiritin, licochalcone A, licochalcone B, and licochalcone C (Table 9).

## 4. Conclusions

In this study, the HPLC spectrum-effect relationships of the licorice flavonoids for samples grown in 21 areas in China were studied by GRA and PLS regression. Five components of licorice have the largest contributions to the heat clearing and detoxification effects and phlegm elimination and cough relief effects of licorice. These components could be used for quality control of licorice. Our results provide a data basis for further identification of common peaks and a method for improvement of licorice quality control and drug efficacy evaluations.

## Figures and Tables

**Table 1:** *Glycyrrhiza uralensis* Fisch. sample source information table.

No.	Origin	Growth years
S1	Weizhou, Tongxin County, Wuzhong City, Ningxia	Biennial
S2	Lejing Township, Yanchi County, Wuzhong City, Ningxia	Biennial
S3	Gaoshawo Town, Yanchi County, Wuzhong City, Ningxia Hui Autonomous Region	Biennial
S4	Hongsibao District, Wuzhong City, Ningxia	Biennial
S5	Zhaolou Village, Longde County, Guyuan City, Ningxia	Biennial
S6	Shenlin Village, Longde County, Guyuan City, Ningxia	Biennial
S7	Yuzhong County, Lanzhou City, Gansu Province	Biennial
S8	Yongdeng County, Lanzhou City, Gansu Province	Biennial
S9	Changning Town, Minqin County, Wuwei City, Gansu Province	Biennial
S10	Qingshui Township, Min County, Dingxi City, Gansu Province	Biennial
S11	Longxi County, Dingxi City, Gansu Province	Biennial
S12	Xifeng District, Qingyang City, Gansu Province	Biennial
S13	Huining County, Baiyin City, Gansu Province	Biennial
S14	Songshan District, Chifeng City, Inner Mongolia	Biennial
S15	Wanniute Banner, Chifeng City, Inner Mongolia	Biennial
S16	Hangjin Banner, Ordos City, Inner Mongolia	Biennial
S17	Linxian County, Lvliang City, Shanxi Province	Biennial
S18	Alar, Xinjiang	Biennial
S19	Horgos Port, Yili Kazakh Autonomous Prefecture, Xinjiang	Biennial
S20	Garsu Village, Horgos City, Yili Kazakh Autonomous Prefecture, Xinjiang	Biennial
S21	Xiangfang District, Harbin City, Heilongjiang Province	Biennial

**Table 2:** The standards of licorice information table.

Name of standards	CAS	Batch number
Liquiritin	551-15-5	Z10J8X39611
Isoliquiritin	5041-81-6	R07D8F50056
Liquiritigenin	578-86-9	Z20J8X40265
Isoliquiritigenin	961-29-5	R12A7F19390
Liquiritin apioside	74639-14-8	Y16D7Y306709
Isoliquiritin apioside	120926-46 -7	P13A9F58700
Neoliquiritin	5088-75-5	Y18A7H19583
Neoisoliquiritin	7014-39-3	Y08J8H37579
Glabridin	59870-68-7	G006171216
Licochalcone A	58749-22-7	P21O8F46473
Licochalcone B	58749-23-8	P15D6F7553
Licochalcone C	144506-14-9	P13A9F58701
Formononetin	485-72-3	16031005
Glycyrrhizic acid	1405-86-3	P11A9F58301

**Table 3:** Similarity analysis of fingerprint of total flavonoids of *Glycyrrhiza uralensis* Fisch. from 21 producing areas.

No.	Similarity
S1	0.949
S2	0.968
S3	0.956
S4	0.981
S5	0.950
S6	0.945
S7	0.941
S8	0.741
S9	0.954
S10	0.954
S11	0.973
S12	0.617
S13	0.980
S14	0.950
S15	0.906
S16	0.735
S17	0.906
S18	0.956
S19	0.735
S20	0.787
S21	0.959

**Table 4:** Grey correlation between common peaks of flavonoids from *Glycyrrhiza uralensis* Fisch. and phenol red test in mouse trachea.

Peak number	Similarity
1	0.4529
2	0.5355
3	0.6313
4	0.6361
5	0.6170
6	0.6383
7	0.5820
8	0.7090
9	0.6676
10	0.5455
11	0.5814
12	0.6494
13	0.5977
14	0.6504
15	0.6419

**Table 5:** Grey correlation between the common peaks of flavonoids from *Glycyrrhiza uralensis* Fisch. and ammonia-induced cough test.

Peak number	Similarity
1	0.5822
2	0.4822
3	0.6050
4	0.6160
5	0.6050
6	0.5701
7	0.5898
8	0.6709
9	0.5905
10	0.5425
11	0.5637
12	0.5987
13	0.5680
14	0.6375
15	0.5281

**Table 6:** Grey Correlation between the common peaks of flavonoids from *Glycyrrhiza uralensis* Fisch. and SO_2_-induced cough test.

Peak number	Similarity
1	0.5338
2	0.4392
3	0.6082
4	0.6344
5	0.6389
6	0.5801
7	0.5903
8	0.6251
9	0.6521
10	0.5776
11	0.5923
12	0.6457
13	0.5704
14	0.6493
15	0.6572

**Table 7:** Grey correlation between the common peaks of flavonoids from *Glycyrrhiza uralensis* Fisch. and foot swelling test.

Peak number	Similarity
1	0.5220
2	0.4196
3	0.6303
4	0.6509
5	0.6372
6	0.6136
7	0.6125
8	0.7337
9	0.6064
10	0.5721
11	0.5920
12	0.6547
13	0.5773
14	0.6431
15	0.6353

**Table 8:** Grey correlation between the common peaks of flavonoids from *Glycyrrhiza uralensis* Fisch. and xylene-induced ear swelling in mice.

Peak number	Similarity
1	0.4001
2	0.5457
3	0.6575
4	0.6488
5	0.6957
6	0.6404
7	0.5959
8	0.7537
9	0.6366
10	0.5245
11	0.6179
12	0.6249
13	0.5887
14	0.6548
15	0.7016

**Table 9:** Assignment of common peaks in fingerprint of flavonoids in *Glycyrrhiza uralensis* Fisch. based on HPLC-Q-TOF/MS.

Peak number	Name	Formula	RT	m/z	Mass	Score	Theoretical isotopic
3	Liquiritin apioside	C_26_H_30_O_13_	3.954	550.8892	550.5086	99.48	551.1700 (24.42%)
5	Neoisoliquiritin	C_21_H_22_O_9_	4.185	418.6043	417.1261	99.12	419.1272 (20.87%)
6	Licochalcone B	C_16_H_14_O_5_	4.745	286.1567	285.0841	98.12	287.0843 (14.79%)
14	Licochalcone C	C_21_H_22_O_4_	8.056	338.4099	337.3969	98.30	339.1501 (20.21%)
15	Licochalcone A	C_21_H_22_O_4_	8.332	338.4094	337.4039	98.63	339.1532 (12.53%)

## Data Availability

The data used to support the findings of this study are included within the article.

## Supplementary Materials

Supplementary Materials Preparation of sample solutions and method validation for quantitative analysis of total flavonoids content.

## References

[1] Wang Y., Yang Y. (2007). Simultaneous quantification of flavonoids and triterpenoids in licorice using HPLC. *Journal of Chromatography B*.

[2] China Medical Science (2015). *Pharmacopoeia of People’s Republic of China*.

[3] Li X., Chen L., Li G. (2013). Ecological distribution and propagative technique research of *Glycyrrhiza* resources in China. *Ecology and Environmental Sciences*.

[4] Zhang Q., Ye M. (2009). Chemical analysis of the Chinese herbal medicine Gan-Cao (licorice). *Journal of Chromatography A*.

[5] Mamedov N. A., Egamberdieva D. (2019). Phytochemical constituents and pharmacological effects of licorice: a review. *Plant and Human Health*.

[6] Bahmani M., Rafieian-Kopaei M., Karamati S. A. (2014). A review of the health effects and uses of drugs of plant licorice (*Glycyrrhiza glabra* L.) in Iran. *Asian Pacific Journal of Tropical Disease*.

[7] Lee J. H., Ze K. R., Kim D.-H. (2009). Analysis of liquiritigenin, an aglycone of liquiritin in licorice by high performance liquid chromatography. *Korean Journal of Pharmacognosy*.

[8] Ju S. M., Kim M. S., Jo Y. S. (2017). Licorice and its active compound glycyrrhizic acid ameliorates cisplatin-induced nephrotoxicity through inactivation of p53 by scavenging ROS and overexpression of p21 in human renal proximal tubular epithelial cells. *European Review for Medical and Pharmacological Sciences*.

[9] Zhu T. T., Wu L., Wang X. L. (2017). Investigation on relationships between chemical spectrum and bioeffect of prepared rhubarb decoction in rats by UPLC-ESI-Q-TOF-MS method coupled with gray correlation analysis. *Journal of Functional Foods*.

[10] Li W., Sun X., Liu B., Zhang L., Fan Z., Ji Y. (2016). Screening and identification of hepatotoxic component in evodia rutaecarpa based on spectrum-effect relationship and UPLC-Q-TOFMS. *Biomedical Chromatography*.

[11] Shi Z., Liu Z., Liu C. (2016). Spectrum-effect relationships between chemical fingerprints and antibacterial effects of *Lonicerae japonicae* flos and *Lonicerae* flos base on UPLC and microcalorimetry. *Frontiers in Pharmacology*.

[12] Pattanayak S. P., Sunita P. (2009). In vivo antitussive activity of *Coccinia grandis* against irritant aerosol and sulfur dioxide-induced cough model in rodents. *Bangladesh Journal of Pharmacology*.

[13] Chen Q. (2011). *Research Methods in Pharmacology of Chinese Materia Medica*.

[14] Hatano T., Takagi M., Ito H., Yoshida T. (1998). Acylated flavonoid glycosides and accompanying phenolics from licorice. *Phytochemistry*.

[15] Jia S. (2016). Research progress of the functions of licorice flavonoids. *Journal of Beijing Union University*.

[16] Xing G., Li N., Wang T., Yao M.-Y. (2003). Advances in studies on flavonoids of *Licorice*. *China Journal of Chinese Materia Medica*.

[17] Qiao X., Liu C.-F., Ji S., Lin X.-H., Guo D.-A., Ye M. (2014). Simultaneous determination of five minor coumarins and flavonoids in Glycyrrhiza uralensis by solid-phase extraction and high-performance liquid chromatography/electrospray ionization tandem mass spectrometry. *Planta Medica*.

[18] Liu X., Li Q., Lv C. (2015). Combination of the advantages of chromatographic methods based on active components for the quality evaluation of licorice. *Journal of Separation Science*.

[19] Liu S., Lin Y. (2011). Introduction to Grey systems modeling software. *Understanding Complex Systems*.

[20] Zhang X., Chen J., Yang J.-L., Shi Y.-P. (2017). UPLC-MS/MS analysis for antioxidant components of Lycii fructus based on spectrum-effect relationship. *Talanta*.

[21] Zhao Y., Liu S.-X., Zhang C.-X., Liu D.-L. (2016). Analysis on chemical constituents from *Glycyrrhizae* radix et Rhizoma by HPLC-Q-TOF-MS. *Chinese Traditional & Herbal Drugs*.

