# Supplementary material for: Spectrum-Effect Relationships of Flavonoids in Glycyrrhiza uralensis Fisch
Source: J Anal Methods Chem. 2020 Dec 3;2020:8838290. doi: 10.1155/2020/8838290 (PMC7728469; doi:10.1155/2020/8838290)
Supplement: Supplementary Materials — Preparation of sample solutions and method validation for quantitative analysis of total flavonoids content. [file 8838290.f1.doc]

*1.Method validation for quantitative analysis of total flavonoids content*

Five sample solutions from the same batch were analyzed to determine the precision. The repeatability was evaluated by analyzing one sample five times. Liquiritin standard was added to nine sample solutions of flavonoids from the same batch and absorbance value was determined by UV spectrophotometry. Each method validation was performed three times in parallel.

*2 Preparation of total flavonoids content*

A 1000-g sample of each licorice was passed through a 60 mesh sieve, dried at 60°C to a constant mass, and then boiled three times (1 h each) in a volume of water five times the sample mass. After filtration, 60% ethanol was added to the filtrate and it was left overnight at room temperature. Next, the supernatant was filtered, and the filtrate was reduced under vacuum to remove the alcohol and obtain the licorice extract. Each licorice extract was submitted to polyamide column chromatography with a progression from 80–100 mesh and eluted sequentially with distilled water, 30% ethanol, and 70% ethanol elution until the elute was colorless. The eluent was then fractionated to obtain water, 30% ethanol, and 70% ethanol fractions. The 70% ethanol fraction was reduced under vacuum and dried to obtain the licorice total flavonoids. Samples of the licorice flavonoids concentrates were weighed to 30 mg in 10-mL volumetric flasks and 70% ethanol was added. After ultrasonication for 30 mins, each sample was weighed and then stored at 4°C until required for analysis.

*3 Validation of the fingerprint analysis*

One sample solution was analyzed six times to determine the precision. A stability study was performed by analyzing a sample at different intervals over 1 day (0, 2, 4, 8, 12, and 24 h). Six sample solutions from the same batch were analyzed to determine the repeatability. The analysis of each sample was repeated three times.All the results show that the method has good precision, stability and repeatability.
